# Indifference of Southern Physicians to Their Home Journals

**Published:** 1884-11-20

**Authors:** 


### *INDIFFERENCE OF SOUTHERN PHYSICIANS TO THEIR HOME JOURNALS.*

In respect to circulation and popularity, we believe we are second to no journal in the South, yet, as Southern journalists, we have to admit the truth of a remark once made by a medical editor in the Georgia Medical Association, when discussing the causes of the repeated failures of medical publications in the South. He said : "*Southern doctors take Northern journals, but Northern doctors will not take Southern journals.*" We make an exception as to the Northwest, where our list is growing.

Why this is so is difficult to answer. It cannot be said it is because of the inferiority of Southern journals, or a want of ability on the part of Southern writers. On the contrary, the contributions of Southern practitioners are usually more practical, and certainly better adapted to the wants of the Southern practitioner, than those of Northern writers. But we have to complain that some of our ablest writers send their articles to the North for publication, where, often, they attract little attention, and do no good.

In this connection it is appropriate to refer to a letter just received by a medical friend in Louisiana, inquiring to know in what Northern journal a certain paper was published on Hemorrhagic Malarial Fever. He desired to see the article, because he had many cases of the disease to treat, and wanted all the light he could get on the subject. The author of the article referred to was a practitioner of Arkansas.

We will not draw the moral from this fact, which is patent to every one.— We will only take this occasion to say to our subscribers : Sustain your home journals, write for them, and encourage them in every possible manner. Time for renewals to the RECORD is close at hand. Don't fail to renew, and, in addition, send up a new subscriber with your own name, and thus help us to increase our list.

To any one sending us a new subscriber on renewal, we will allow 50 cents off of each name ; or, for $3 we will send the RECORD to yourself and the new name which you send us for 1885.

